# Clinicopathologic significance and prognostic value of Ki-67 expression in patients with gastric cancer: a meta-analysis

**DOI:** 10.18632/oncotarget.17305

**Published:** 2017-04-20

**Authors:** Guanying Luo, Yunzhao Hu, Zhiqiao Zhang, Peng Wang, Zhaowen Luo, Jinxin Lin, Canchang Cheng, You Yang

**Affiliations:** ^1^ Department of Infectious Diseases, The First People's Hospital of Shunde, Shunde, Guangdong, China; ^2^ Department of Internal Medicine, The Chencun Affiliated Hospital of First People's Hospital of Shunde, Shunde, Guangdong, China

**Keywords:** gastric cancer, ki-67, meta-analysis, prognostic value

## Abstract

**Background:**

The prognostic value and clinicopathologic significance of Ki-67 expression in gastric cancer patients was controversial. This meta-analysis was performed to clarify the prognostic value and clinicopathologic significance of Ki-67 expression in gastric cancer patients.

**Materials and Methods:**

Several electronic databases were searched for eligible studies. The pooled odds ratio (OR), hazard ratios (HR) and 95% confidence interval(CI) were calculated to explore the prognostic value and clinicopathologic significance of Ki-67 expression for disease free survival and overall survival.

**Results:**

Totally 5600 gastric cancer patients from 29 studies were included in this study. High Ki-67 expression was significantly related with Lauren's classification (OR = 1.70; *P* = 0.001; 95%CI: 1.40-2.06) and tumor size(OR = 1.54; *P* = 0.006; 95%CI: 1.14-2.09). However, high Ki-67 expression was not significantly associated with lymph node metastasis (OR = 1.37; *P* = 0.138; 95% CI: 0.90-2.08), tumor stage (OR = 1.31; *P* = 0.296; 95% CI: 0.79-2.16) and tumor differentiation (OR = 1.03; *P* = 0.839; 95% CI: 0.78-1.35). The pooled HRs were 1.87(*P* = 0.001; 95% CI 1.30-2.69) for disease free survival and 1.23(*P* = 0.005; 95% CI 1.06-1.42) for overall survival.

**Conclusions:**

High Ki-67 expression may serve as a predictive biomarker for poor prognosis in gastric cancer patients. Stratification by Ki-67 expression may be a consideration for selection of therapeutic regimen and integrated managements.

## INTRODUCTION

Gastric cancer (GC) is the fourth malignant tumor and the second leading cause of tumor related death in the world [1]. Patients with advanced GC has only a median overall survival (OS) of less than 12 months [2–3]. Therefore, there is an urgent need for reliable prognostic factors to predict poor prognosis and to subdivide different risk stratification for management of GC patients.

Ki-67 is a nuclear protein which expresses throughout the cell cycle in proliferating cells [4]. The correlation between Ki-67 expression and prognosis of GC patients were still contradictory [5–33]. Meanwhile, the clinicopathologic significance of Ki-67 expression in GC patients was uncertain. Therefore, we performed this meta-analysis to determine the clinicopathologic significance and prognostic value of Ki-67 expression in GC patients.

## RESULTS

### Search results

**Figure 1:**
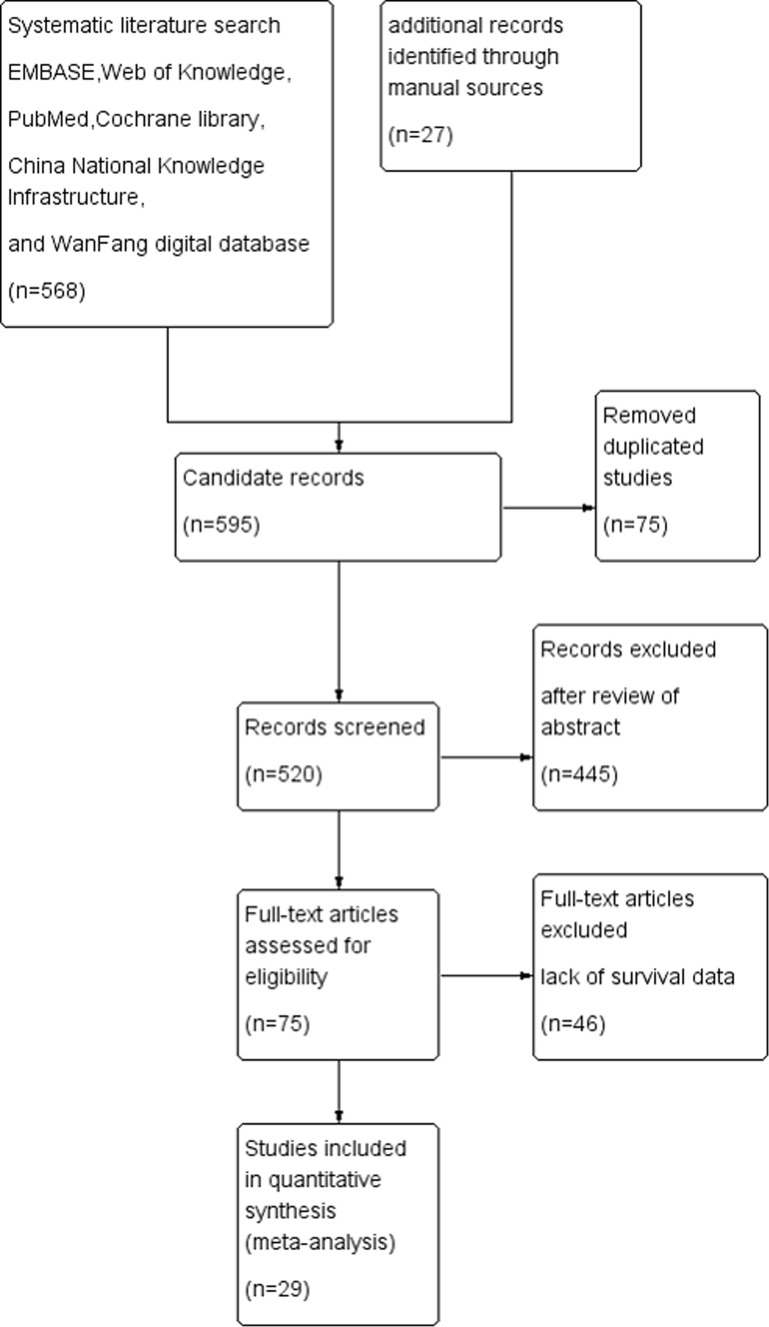
Flowchart of study selection in present meta-analysis

The initial search returned 595 articles (with 75duplicate articles). After screening the abstracts, 445 irrelevant articles were excluded. Reviewers identified 75 potential studies for full-text review and 46 articles were eliminated due to inadequate data. Finally, 29 studies were included in the present study [5–33]. The details of screening process were shown in Figure 1.

### Study selection and characteristics

The characteristic of included studies were summarized in Table 1. The publication time of included studies was between 1996 and 2016. The study sample size was between 56 and 693, with a mean sample size of 193. The NOS score of 29 studies varied from 7 to 8, with a mean value of 7.73. Twenty-three studies provided survival information and fifteen studies presented clinicopathologic parameters.

**Table 1:** Characteristics of studies included in the meta analysis

Study	Type	Method	Cell	Cutoff point	Number	HR	95%CI Lower	95%CI upper	*P* value	NOS score
Muller et al 1996[5]	GC	WTS	1000	53.30%	418	1.04	0.8	1.33	0.77	8
Victorzon et al 1996[6]	GC	WTS	1000	30%	237	1.1	0.78	1.55	0.587	8
Ohtani et al 1998[7]	GC	WTS	NR	35.90%	225	1.307	0.777	2.199	0.313	8
Manzoni et al 1998[8]	GC	WTS	1000	10%	56	NR	NR	NR	NR	7
Ikeguchi et al 1999[9]	GC	WTS	1000	18%	97	1.023	0.994	1.055	0.121	8
Liu et al 2001[10]	GC	WTS	1000	27%	190	NR	NR	NR	NR	7
Al-Moundhri et al 2005[11]	GC	WTS	1000	25%	121	NR	NR	NR	NR	7
Takahashi et al 2006[12]	GC	WTS	1000	50%	122	0.99	0.48	2.05	0.978	8
Takahashi et al 2009[13]	GC	WTS	1000	25%	71	1.25	0.55	2.86	0.594	8
Tsamandas et al 2009[14]	GC	WTS	NR	5%	110	2.93	1.69	5.08	0.001	8
Tzanakis et al 2009[15]	GC	WTS	NR	35%	93	1.48	0.86	2.54	0.157	7
Li et al 2009[16]	GC	WTS	1000	10%	336	2.55	1.8	3.62	0.001	8
Lazar et al 2010[17]	GC	WTS	500	45%	61	1.07	0.62	1.84	0.808	8
Lee et al 2010[18]	GC	WTS	300	10%	245	0.561	0.38	0.83	0.004	8
Zhao et al 2010[19]	GC	WTS	1000	50%	336	NR	NR	NR	NR	7
Ichinoe et al 2011[20]	GC	WTS	1000	40%	87	0.907	0.532	1.547	0.720	8
Nakashima et al 2011[21]	Adenocarcinoma	WTS	1000	48.10%	100	NR	NR	NR	NR	7
Wen et al 2011[22]	GC	TMAS	1000	10%	264	2.56	1.39	5.62	0.003	8
Giaginis et al 2011[23]	GC	WTS	1000	50%	66	NR	NR	NR	NR	7
He et al 2012[24]	GC	TMAS	1000	25%	166	1.85	1.3	2.63	0.001	8
Kang et al 2013[25]	GC	WTS	NR	10%	458	0.63	0.37	1.08	0.089	8
Liu et al 2013[26]	GC	WTS	NR	50%	180	3.44	1.7	6.96	0.001	8
Xiao et al 2013[27]	GC	TMAS	500	1%	43	1.32	0.91	1.9	0.143	8
Yang et al 2014[28]	Adenocarcinoma	WTS	1000	30%	159	1.13	0.63	2.05	0.682	8
Ayed et al 2014[29]	GC	WTS	1000	1%	90	NR	NR	NR	NR	7
Li et al 2015[30]	GC	WTS	NR	50%	69	1.5	0.59	3.71	0.394	8
Boger et al 2016[31]	GC	WTS	500	50%	315	1.08	0.88	1.33	0.461	7
Huang et al 2016[32]	Adenocarcinoma	WTS	NR	50%	693	1.421	1.191	1.695	0.001	8
Ou et al 2016[33]	Adenocarcinoma	TMAS	NR	5%	192	1.1	0.9	1.35	0.352	8

HR, hazard ratio; CI, confidence interval; NOS, Newcastle-Ottawa Quality Assessment Scale.

### Association of Ki-67 expression with clinicopathologic parameters

**Figure 2:**
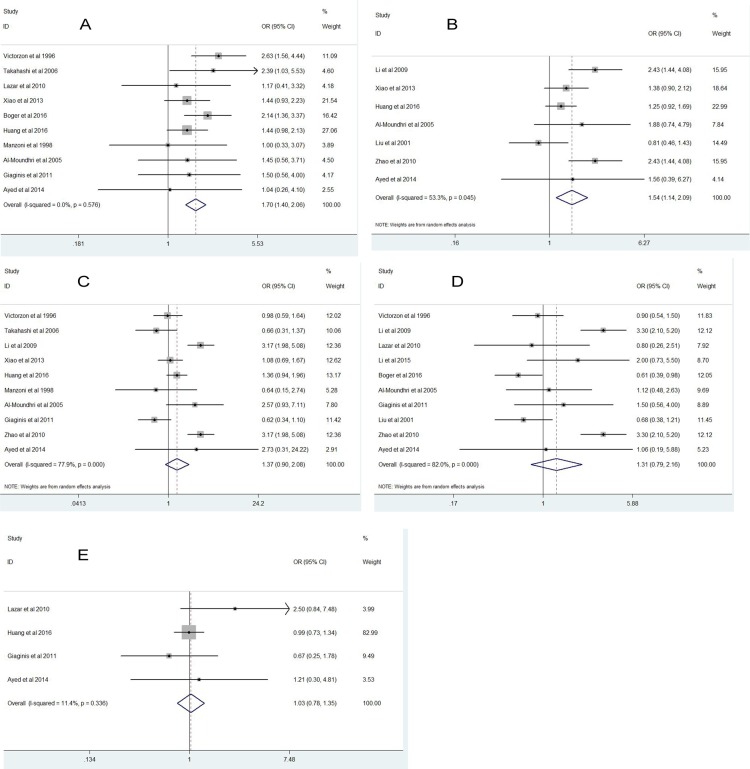
Forest plot diagrams of odds ratios for correlations between Ki-67 expression and pathological parameters

As shown in Figure 2, high Ki-67 expression was significantly related with Lauren's classification (OR = 1.70; *P* = 0.001; 95%CI: 1.40-2.06) and tumor size(OR = 1.54; *P* = 0.006; 95%CI: 1.14-2.09). However, high Ki-67 expression was not significantly associated with lymph node metastasis (OR = 1.37; *P* = 0.138; 95% CI: 0.90-2.08), tumor stage (OR = 1.31; *P* = 0.296; 95% CI: 0.79-2.16) and tumor differentiation (OR = 1.03; *P* = 0.839; 95% CI: 0.78-1.35).

### Prognostic value of high Ki-67 expression in gastric cancer patients

**Figure 3:**
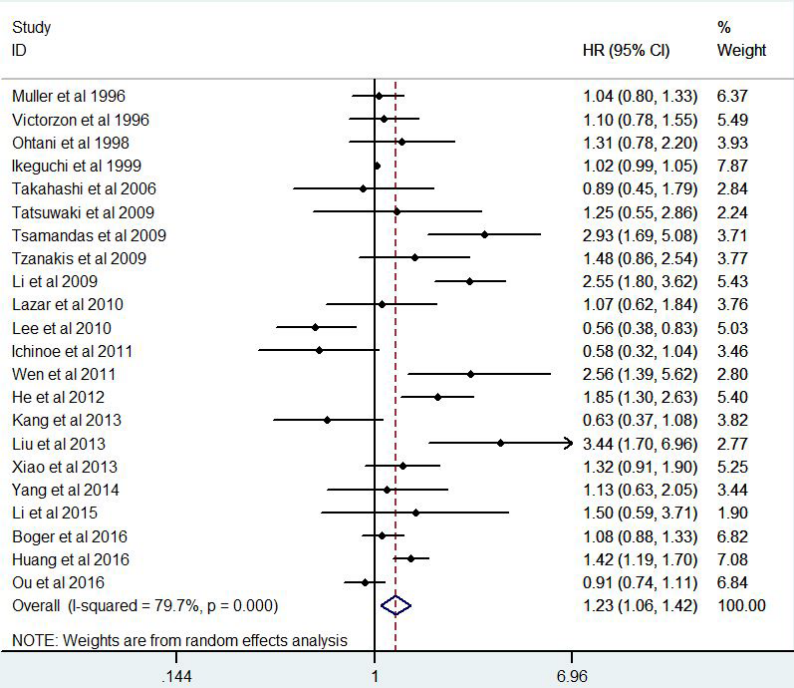
Forest plot diagrams of hazard ratios for correlations between Ki-67 expression and overall survival

**Figure 4:**
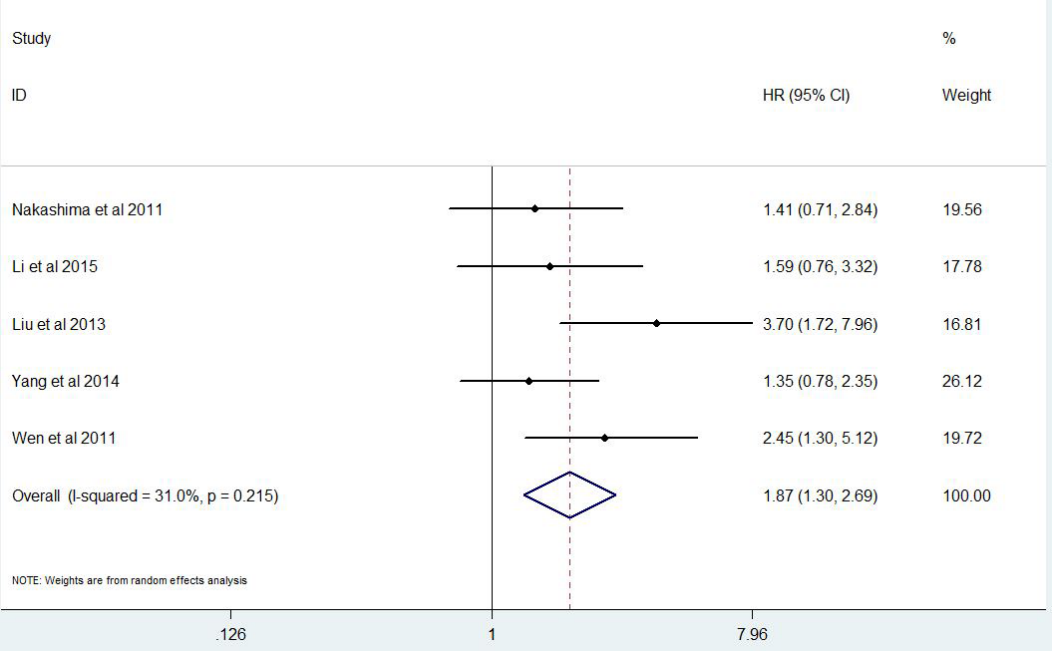
Forest plot diagrams of hazard ratios for correlations between Ki-67 expression and disease free survival

A total of 4741 GC patients from 23 eligible studies were included and analyzed for prognostic value of Ki-67 expression in GC patients(Figure 3and Figure 4). The pooled HRs was 1.87(*P* = 0.001; 95% CI 1.30-2.69) for DFS and 1.23(*P* = 0.005; 95% CI 1.06-1.42) for OS.

### Sensitivity analysis

All studies were sequentially removed to explore that whether any individual study had an significant influence to the pooled HR. The pooled HR in sensitivity meta-analysis ranged from 1.17(95%CI: 1.02-1.34) to 1.28 (95%CI: 1.11-1.48) for OS, demonstrating that the pooled HR was not significantly affected by any individual study(Table 2).

**Table 2:** Effect of individual studies on the pooled HRs of Ki-67 expression for overall survival

Study omitted	Estimate HR	95%CI	
		Lower	Upper
1	1.2472792	1.0675433	1.4572761
2	1.240342	1.0646844	1.4449806
3	1.2284332	1.0576263	1.4268255
4	1.2592941	1.0504285	1.5096903
5	1.243209	1.0714135	1.4425511
6	1.2308739	1.0614478	1.4273434
7	1.1883517	1.03158	1.3689484
8	1.2223002	1.0531162	1.4186638
9	1.1733439	1.0239507	1.3445334
10	1.238404	1.0660043	1.4386851
11	1.2813864	1.1075524	1.4825042
12	1.2639937	1.0909787	1.4644465
13	1.2044084	1.0418602	1.3923169
14	1.2009488	1.0375293	1.3901081
15	1.2637651	1.0899976	1.4652347
16	1.1940765	1.0359601	1.376326
17	1.2269805	1.0546938	1.4274106
18	1.2353078	1.063794	1.4344743
19	1.2264266	1.0582221	1.4213671
20	1.2459158	1.0632767	1.4599268
21	1.2191019	1.0452053	1.4219306
22	1.2612625	1.0773629	1.4765527
Combined HR	1.2309021	1.0641824	1.4237409

HR, hazard ratio; CI, confidence interval.

### Publication bias

**Figure 5:**
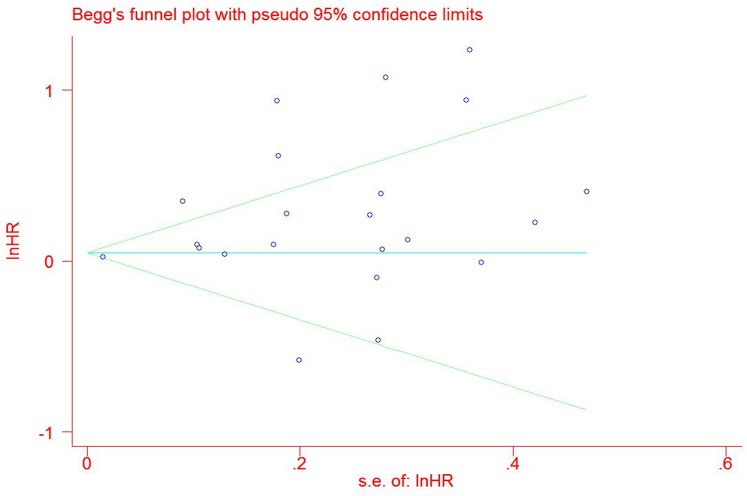
Begg's funnel plot for studies which provided hazard ratios of high Ki-67 expression for overall survival (*P* = 0.499)

The figure of Begg's funnel plot (Figure 5) did not show any evidence of asymmetry for OS (*P* = 0.499). Similarly, there were no evidences for publication bias in terms of Lauren's classification (*P* = 0.721), tumor size(*P* = 0.881), lymph node metastasis (*P* = 0.788), tumor stage (*P* = 1.0) and tumor differentiation (*P* = 0.734). The further Egger's linear regression test did not find any significant evidences of publication bias for Lauren's classification (*P* = 0.435), tumor size(*P* = 0.586), lymph node metastasis (*P* = 0.750), tumor stage (*P* = 0.627), tumor differentiation (*P* = 0.652) and OS(*P* = 0.066).

### Stability assessment of the pooled hazard ratios of Ki-67 expression for overall survival by cumulative meta-analysis

**Figure 6:**
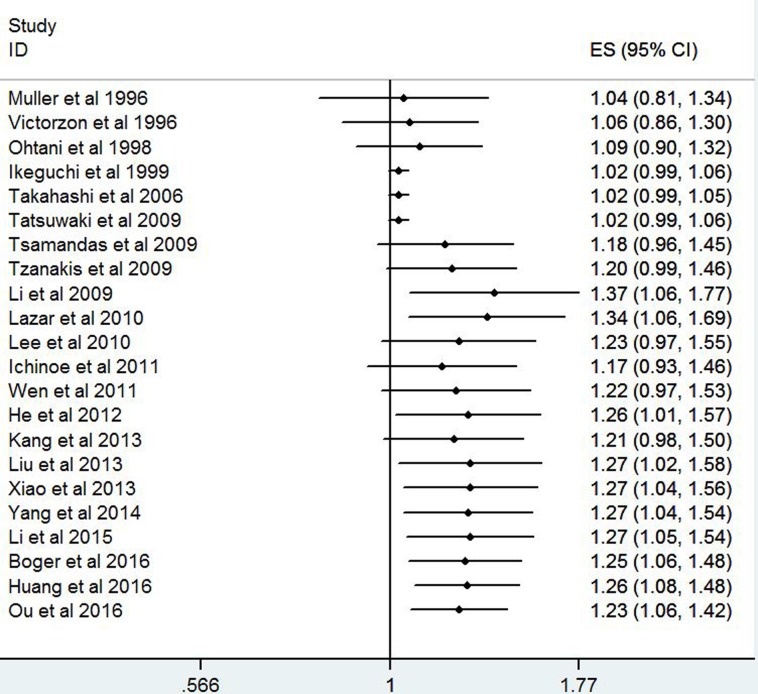
Cumulative meta-analysis for stability of the hazard ratios of Ki-67 for overall survival in gastric cancer patients

The pooled HRs of cumulative meta-analysis(Figure 6) ranged from 1.23(95%CI: 1.06-1.42) to 1.27 (95%CI: 1.04-1.56) for OS since 2013, demonstrating that performance of Ki-67 expression for OS in GC patients was stable and reliable.

### Explore of sources of heterogeneity by meta-regression analyses and subgroup analyses

The pooled HR was 1.31(95% CI 1.05-1.62; *P* = 0.017; heterogeneity = 82.7%; *P* < 0.001) in studies with patient number more than 100 for OS whereas it was 1.08(95% CI 0.91-1.28; *P* = 0.386; heterogeneity = 26.3%; *P* < 0.228) in studies with patient number not more than 100(Table 3). The results suggested that sample size might contributed to the clinical heterogeneity. However, meta-regression analysis did not find any source of heterogeneity (all *P* > 0.05, data not shown).

**Table 3:** Subgroup analyses for associations between Ki-67 expression and overall survival in gastric cancer patients

			Overall survival		95%CI		Heterogeneity	
Group factors	Subgroup	Study	HR	P value	Lower	Upper	*I*^2^	*P* value
Total	Total	22	1.23	0.005	1.06	1.42	79.7%	0.001
patients≥100	Yes	15	1.31	0.017	1.05	1.62	82.7%	0.001
	No	7	1.08	0.386	0.91	1.28	26.3%	0.228
Adenocarcinoma	Yes	3	1.14	0.476	0.80	1.62	81.3%	0.005
	No	19	1.26	0.01	1.06	1.50	80.0%	0.001
THAS	Yes	3	1.45	0.096	0.94	2.25	83.1%	0.001
	No	19	1.19	0.037	1.01	1.41	79.7%	0.001
Cell number=1000	Yes	10	1.27	0.057	0.99	1.62	81.0%	0.001
	No	4	0.97	0.847	0.69	1.36	73.2%	0.011
Cut-off point≥25%	Yes	14	1.24	0.01	1.05	1.46	53.0%	0.01
	No	8	1.24	0.143	0.93	1.66	88.8%	0.001

HR, hazard ratio; CI, confidence interval.

## DISCUSSION

The current meta analysis showed that high Ki-67 expression was significantly related with Lauren's classification (OR = 1.70; 95%CI: 1.40-2.06) and tumor size (OR = 1.54; 95%CI: 1.14-2.09). The pooled HRs were 1.87(95% CI 1.30-2.69) for DFS and 1.23(95% CI 1.06-1.42) for OS. These results demonstrated that high Ki-67 expression significantly predicts poorer prognosis compared with low Ki-67 expression.

Some previous studies have reported that high Ki-67 expression was associated with poor OS in GC patients[14, 16, 22, 24, 26, 32]. These original studies have revealed that high Ki-67 expression had a predictive value for prognosis of GC patients. Our conclusion was consistent with that of these previous studies. Recently, several meta analyses have reported that high Ki-67 expression was associated with poor prognosis in different tumors, including gastrointestinal stromal tumor, cervical cancer and non-small cell lung cancer [35–37]. Furthermore, two studies have further reported that Ki-67 expression could be used for risk stratification in patients with gastrointestinal stromal tumor [38–39].

The heterogeneity was significant in the present meta-analysis. There might be some potential sources of heterogeneity as follows: First, the heterogeneity caused by different cut-off values of Ki-67 expression was inevitable. Second, subgroup analyses showed that sample size might be a potential source of heterogeneity. Third, the identify methods of Ki-67 expression (TMAS or WTS) and the number of count cells(1000 or 500) might yield variation in different studies. In addition, heterogeneity could be caused by other factors, such as study regions, pathology types, tumor stages, treatments and races.

Although significant heterogeneity existed in the present meta-analysis, sensitivity analyses and cumulative meta-analyses demonstrated that HRs of Ki-67 expression for prognosis of GC patients was stable and reliable. Furthermore, we performed Begg's funnel plot and Egger's test to assess the potential publication bias and did not find any evidence of publication bias.

The present meta analysis had several strengths: Firstly, we first explored the association between Ki-67 expression and clinicopathologic parameters in GC patients. Secondly, we included 29 eligible studies and 5600 patients, which could strengthen persuasiveness of the conclusions. Thirdly, Ki-67 expression in 29 eligible studies was all detected by IHC. Fourthly, studies published in Chinese were included as English literature to increase representation of study population.

The results of the present meta analysis need to be interpreted cautiously for several limitations. First, most studies defined positive status of Ki-67 expression according to different cut-off values. Second, heterogeneity was inevitable due to different baseline characteristics. Third, although the method for extracting survival information from survival curve is widely accepted, we could not completely eliminate the sources of information inaccuracy in the process of extracting data.

In conclusion, high Ki-67 expression may serve as a predictive biomarker for poor prognosis in gastric cancer patients. Stratification by Ki-67 expression may be a consideration for selection of therapeutic regimen and integrated managements.

## MATERIALS AND METHODS

### Search strategy

Several electronic databases, including PubMed, EMBASE, Cochrane Library, Web of Knowledge, China National Knowledge Infrastructure and WanFang data, were searched from January 1970 to May 2016. We performed literature search by combined text word and MeSH(Emtree for EMBASE database accordingly) strategy with terms “ Ki-67 Antigen” or “MIB-1 Antigen” and “gastric cancer” or “gastric carcinoma” or “stomach tumor” and “survival” or “outcome” or “prognosis” or “prognostic”. The strategy was correspondingly adjusted in different databases. In the retrieval process, expanded search of hyponym was performed. We made a manual search using the reference lists of the relevant articles. We contacted the corresponding author to get necessary information if necessary. The search was restricted to human studies, but there was no restriction on language or publication time. All clinical investigation and data achievement were conducted according to the principles expressed in the Declaration of Helsinki.

### Criteria for inclusion and exclusion

The inclusion criteria were as follows: (1) proven pathological diagnosis of GC in humans; (2) Ki-67 expression evaluation using immunohistochemistry (IHC) method; (3)provided information on clinicopathological parameters and/or overall survival information. Studies not directly providing survival information were included if survival information were available from survival curve. Articles published in Chinese were included as English literature. Only the most recent study was included among duplicate studies. There were no restrictions on sample size or follow-up period.

The following studies were excluded: (1) reviews, letters, case reports, and conference abstracts without original data; (2) non-human experiments; (3) laboratory studies; (4) articles from which the necessary information could not be extracted.

### Quality assessment of studies

Two reviewers (Zhiqiao Zhang and Jinxin Lin) independently assessed the quality of studies using the Newcastle-Ottawa Quality Assessment Scale(NOS) (Table 1). Disagreements were resolved through consensus with a third reviewer (Guanying Luo).

### Data extraction

Two investigators (Zhiqiao Zhang and Jinxin Lin) independently extracted and examined the following data: surname of the first author, publication year, country, sample size, disease stage, detection method of Ki-67, clinical parameters and survival outcome data. Study information was extracted and recorded using a standardized form. All eligible studies were coded as surname of the first author + publish year in the standardized form. Study authors were contacted to obtain key information if necessary. When necessary, a third investigator (Guanying Luo) helped to reach a consensus.

### Statistical analysis

The statistical analysis was performed according to the guidelines suggested by the Meta-Analysis of Observational Studies in Epidemiology group(MOOSE)[34]. The pooled odds ratio (OR) were combined to explore the association between KI-67 expression and clinicopathological parameters. The pooled hazard ratio (HR) were used to summary outcome of overall survival. While survival data were not directly reported, we extracted survival information from Kaplan-Meier curve. The heterogeneity among different studies was measured by the Q and *I*^2^ tests. A probability value of *I*^2^ ≥30% and *P* < 0.1 indicated the existence of significant heterogeneity. A random effect model (DerSimonian and Laird method) or fixed effect model(Mantel-Haenszel method) was used depending on the results of heterogeneity analysis. The potential publication bias was assessed by Begg's funnel plot and Egger's test. *P* value < 0.05 was considered statistically significant. The statistical analyses were performed by STATA version 12.0 software (Stata Corporation, College Station, Texas, USA).

## Abbreviations

GC: Gastric cancer
OR: odds ratio
HR: hazard ratio
CI: confidence interval
